# Synthesis of Palladium Nanowires on Flagella Template for Electrochemical Biosensor Detection of microRNA-21

**DOI:** 10.3390/biology13120960

**Published:** 2024-11-22

**Authors:** Kuo Yang, Jueyu Wang, Ying Zhang, Daizong Cui, Min Zhao

**Affiliations:** College of Life Science, Northeast Forestry University, Harbin 150000, China; yangkuo9593@163.com (K.Y.); 18804503512@163.com (J.W.); 18800481223@163.com (Y.Z.)

**Keywords:** flagella extraction, palladium nanowires, biotemplating, electrochemical biosensors, microRNA-21 assay

## Abstract

In recent years, the use of bacterial flagella as biomimetic templates has gained increasing attention in nanomaterial synthesis due to their unique structural and functional properties. In this study, we optimized the flagella extraction method and achieved a high concentration of flagella solution. Flagella were isolated from *Escherichia coli*. Surface characterization revealed that the flagella had abundant functional groups, such as amino and carboxyl groups, which can serve as nucleation sites for the controlled nucleation and growth of metal nanomaterials. Using bacterial flagella as a template, we synthesized one-dimensional palladium nanowires (Fla-Pd NWs). The results of morphological and phase analyses showed that the synthesized palladium nanoparticles were uniformly and densely distributed on the surface of the flagella. Moreover, the Fla-Pd nanowires exhibited superior electrocatalytic activity, which was applied to develop an electrochemical biosensor. This biosensor was used to detect the early breast cancer biomarker microRNA-21 and exhibited a linear range of 0.66–1.98 µmol/L and a detection limit of 0.78 µmol/L. The method demonstrated high selectivity and reusability, making it a promising strategy for early cancer diagnosis.

## Simple Summary

This study proposed a new method for extracting *E. coli* flagella, which served as a template for synthesizing palladium (Pd) nanowires. By improving the flagella extraction process, we successfully produced Pd nanowires with better structure and function, which could have important applications in technology and medicine. Traditional methods often led to impurities that interfered with nanomaterial synthesis, but our method provided a cleaner and more efficient way of producing high-quality nanowires. Using *E. coli* flagella as a template offered environmental benefits, as it eliminated the need for toxic chemicals and reduced energy consumption. This eco-friendly approach was also safer and more reliable for practical applications. Our research also showed how the functional groups on the flagella surface helped form strong bonds with metals, improving the nanowire formation process. This study highlighted the potential of using biological templates for nanomaterial synthesis, contributing to the development of greener and more efficient technologies.

## 1. Introduction

MicroRNAs (miRNAs) are small noncoding RNA molecules that regulate gene expression. By targeting messenger RNAs (mRNAs) for degradation and repression of translation, miRNAs influence various cellular processes, including development, differentiation, proliferation, and apoptosis [1]. MiRNAs play regulatory roles in various diseases, most notably cancer, where they play dual roles as oncogenes and tumor suppressors depending on the cellular context and target gene networks [2].

MicroRNAs contribute significantly to tumorigenesis through their ability to modulate key signaling pathways and cellular mechanisms [3,4]. At the transcription level, miRNAs can inhibit mRNA translation, while at the protein level, they often bind to the 3′-untranslated regions (3′-UTRs) of mRNAs, leading to mRNA degradation and downregulation of target genes [5,6]. This multifaceted regulatory ability of miRNAs highlights their importance in maintaining cellular homeostasis and their dysregulation in cancer progression [7].

Among the various miRNAs studied, miRNA-21 is important for its robust oncogenic properties and extensive involvement in several types of cancers, including colorectal, pancreatic, gastric, and breast cancers [8,9]. The overexpression of miRNA-21 is associated with enhanced tumor growth, metastasis, and poor prognosis; thus, it is a valuable biomarker for cancer diagnosis and prognosis [10]. The stability of miRNA-21 in serum further facilitates its use in noninvasive cancer diagnosis, providing a reliable means for early detection [11].

Although miRNAs are promising, their detection and quantification present significant technical challenges. Traditional methods such as Northern blotting, microarray analysis, and quantitative reverse transcription PCR (qRT-PCR) not only have low sensitivity and throughput but also have operational complexity and adverse environmental effects [9,12]. The short length, low abundance, and high sequence homology of miRNAs make their detection even more difficult, demanding more efficient, biocompatible, and eco-friendly solutions [13]. During the synthesis of nanoparticle-based sensors, heavy metals like HgCl_2_ and AgNO_3_ were frequently employed, which posed significant environmental and biological toxicity [14,15]. Additionally, organic solvents such as CH_2_Cl_2_ and CH_3_CN, commonly used in nanoparticle preparation, presented risks due to their volatility and toxicity [15]. These chemicals, though effective for enhancing sensor performance, introduced safety concerns and environmental hazards. Thus, while these techniques offered high sensitivity and specificity, the emphasis was on the chemical synthesis of biosensors, where the use of toxic reagents raised important considerations regarding safety and environmental sustainability.

Recent advancements in biosensor technologies, particularly electrochemical biosensors, have shown great promise in overcoming the above-mentioned limitations [16]. Electrochemical biosensors offer real-time monitoring capabilities and high analytical sensitivity, making them suitable for quantifying miRNAs in various biological samples [17]. By integrating nanotechnology with electrochemical sensors, researchers have developed electrochemical nanobiosensors, which take advantage of the unique properties of nanomaterials to increase signal transduction and detection sensitivity [18].

Palladium (Pd) nanomaterials, due to their excellent electrical conductivity and catalytic properties, are favorable platforms for biosensing applications [19]. The shape and size of Pd nanomaterials significantly influence their electrocatalytic activity, and various synthesis methods have been developed to produce Pd nanomaterials with optimal activity [20]. However, many traditional synthesis methods require large amounts of organic solvents or surfactants, making them economically and environmentally unfavorable. Therefore, green and cost-effective synthesis methods need to be developed for Pd nanomaterials [21].

Biological templates, such as *E. coli* bacterial flagella, represent a novel approach to synthesizing metal nanomaterials [22]. Due to their biocompatibility and functional group richness, flagella serve as excellent scaffolds for the nucleation and growth of metal nanoparticles. This biotemplating approach not only simplifies the synthesis process but also increases the biocompatibility and functional properties of the resulting nanomaterials [23]. Additionally, the biosensors developed using these nanomaterials demonstrate unique applicability, facilitating specific high-concentration miRNA detection while keeping the process eco-friendly and safe [24].

In this study, we utilized bacterial flagella as biological templates to synthesize Fla-Pd NWs and developed an electrochemical biosensor for miRNA detection. This approach allowed us to not only synthesize a material that has high biocompatibility, functional versatility, and environmental friendliness but also offered a novel pathway for miRNA sensing. The Fla-Pd NWs, characterized by their high surface area and abundant active sites, provide a robust platform for sensitive and label-free detection of miRNAs. This novel biosensing approach, which integrates biotemplated nanomaterials and electrochemical detection, may significantly advance the field of high-concentration miRNA diagnostics. It also provides a framework for optimizing methodologies to meet the stringent requirements of clinical and biomedical research.

## 2. Materials and Methods

### 2.1. Reagents

Tryptone, yeast extract, NaCl, KCl, Na_2_HPO_4_, K_2_HPO_4_, HCl, NaBH_4_, NaOH, and K_3_ [Fe(CN)_6_] were purchased from Sinopharm Chemical Reagent Co., Ltd., Shanghai, China. Toluidine blue (TB) and KBr were obtained from Shanghai Aladdin Biochemical Technology Co., Ltd., Shanghai, China. Ultrafiltration tubes were purchased from Merck & Co., Inc., Kenilworth, New Jersey, USA. Na_2_PdCl_4_, phosphotungstic acid, and DEPC-treated water were purchased from Sigma-Aldrich, St. Louis, Missouri, USA. Lysozymes were purchased from Thermo Fisher Scientific Inc., Waltham, Massachusetts, USA. The Bradford protein assay kit was purchased from TIANGEN Biotech Co., Ltd., Beijing, China. Al_2_O_3_ polishing powder was purchased from Anhui Hesen New Materials Co., Ltd., Bengbu, China.

### 2.2. Extraction of E. coli Flagella

The *E. coli* flagella was extracted using three different methods: acid hydrolysis, traditional mechanical extraction, and the optimized extraction method developed in this study [25,26]. The acid hydrolysis method involves using acid treatment to separate the flagella from bacterial cells, while the traditional mechanical extraction method relies on mechanical agitation to isolate the flagella. The *E. coli* culture was incubated in LB medium at 28 °C and 120 rpm for 24 h until the logarithmic growth phase was reached. The optimized extraction method involved the following steps. First, 6 L of *E. coli* culture was centrifuged at 3500 rpm for 30 min at 4 °C. After removing the supernatant, the bacterial pellet was resuspended in 2 L of pre-chilled sterile PBS buffer and centrifuged again to wash the cells. Next, the pellet was resuspended in autoclaved saline solution and centrifuged at 9000 rpm for 30 min. The supernatant, which contained the flagella, was collected. This flagellum-containing solution was subjected to ultrafiltration using 50 kDa and 100 kDa ultrafiltration tubes to remove salts and concentrate the flagella. The solution obtained after ultrafiltration was the flagella solution.

### 2.3. Synthesis of Fla-Pd NWs

A 10 mM Na_2_PdCl_4_ solution was prepared and dissolved by sonication for 2 h. Then, the solution was left undisturbed for 24 h before use and stored at 4 °C. To synthesize Fla-Pd nanowires (Fla-Pd NWs), 1 mL of the prepared flagella solution was mixed with 200 μL of the 10 mM Na_2_PdCl_4_ solution. The pH of the mixture was adjusted to 7.0 using a 1 M NaOH solution. The mixture was then vortexed for thorough mixing and incubated at 25 °C for 24 h.

After the initial incubation, the pH of the solution was checked and adjusted to 7.0 if necessary, followed by incubation at 25 °C for another 24 h. After incubation, the mixture was centrifuged at 8000 rpm to remove any unbound palladium ions, and the pellet was resuspended in 900 μL of deionized water.

Next, 100 μL of 5 mM NaBH_4_ solution was added dropwise to the flagella solution while gently shaking the centrifuge tube to ensure that the reducing agent was thoroughly mixed. This process resulted in the formation of Fla-Pd NWs, which were stored at 4 °C.

### 2.4. Preparation of E. coli Flagella Samples for TEM Observation

To observe *E. coli* flagella extracted by different methods via transmission electron microscopy (TEM), images were captured by JEM-2100, a JEOL Ltd., Tokyo, Japan instrument. Samples were prepared as follows. First, a 2% phosphotungstic acid (PTA) solution was prepared, and its pH was adjusted to 7 with a 10 mM NaOH solution. A 10 μL aliquot of the flagella suspension was then pipetted onto a 300-mesh carbon grid and allowed to stand for about 10 min. The excess flagella solution around the grid was removed using filter paper, and the sample was allowed to air-dry naturally. A 10 μL aliquot of the 2% PTA solution was applied to the dried sample and allowed to stand for 60 s. The excess staining solution was removed and the sample was again air-dried naturally. The *E. coli* flagella and Fla-Pd NWs were loaded onto the sample holder for TEM examination. High-resolution transmission electron microscopy (HRTEM) by JEM-2100, a JEOL Ltd., Tokyo, Japan instrument, as an integral part of TEM, was utilized to observe the fine structural details of the flagella samples. This high-resolution technique allowed us to capture precise images of the flagella at the atomic level, enhancing the quality and detail of the TEM analysis.

### 2.5. Preparation of the Fla-Pd NW Electrochemical Biosensor

In this experiment, the sequences of nucleic acids used for synthesizing the necessary probes were obtained from previous studies and are listed in Table S1. The capture probe and the microRNA sequences were purchased from Sangon Biotech Co., Ltd., Shanghai, China. The relevant buffers used for these experiments are listed in Table S2. The Fla-Pd NW-modified glassy carbon electrode (GCE) was immersed in 1.0 μM ssRNA (capture probe) in 200 μL of fixation buffer for 1.5 h at room temperature to immobilize the capture probe. After immobilization, the electrode was gently rinsed in PBS to remove any unbound ssRNA probes.

Subsequently, the electrode with immobilized ssRNA was incubated with microRNA-21 in 1.0 × SSC solution at 37 °C for 1 h to allow for hybridization (microRNA-21/ssRNA/Fla-Pd NWs/GCE). After hybridization, the biosensor surface was rinsed with 0.1 × SSC to remove any unbound microRNA-21. The microRNA-21/ssRNA/Fla-Pd NWs/GCE was then immersed in a TB solution (5 mM TB, 0.2 mol/L NaCl, 0.1 mol/L PBS, pH 7.4) for 5 min to allow binding. Finally, the modified electrode was gently rinsed with PBS to remove excess TB, ensuring that only TB molecules intercalated with the hybridized RNA sequence remained on the electrode surface.

### 2.6. Fourier-Transform Infrared (FTIR) Spectroscopy

The flagellar mixture was first frozen overnight at −40 °C. Next, the sample was vacuum freeze-dried at −40 °C using a lyophilizer to obtain a powdered sample. The powdered flagella were then mixed with an appropriate amount of KBr and finely ground. After drying, the flagella of *E. coli* and Fla-Pd NWs were compressed into a pellet for FTIR analysis. The FTIR spectrum of the sample was obtained using a Fourier-transform infrared spectrometer (Nicolet iS20, Thermo Fisher Scientific, Waltham, MA, USA) with a scanning range from 500 to 4000 cm^−1^.

### 2.7. Characterization of Fla-Pd NWs by XPS and XRD

The Fla-Pd NWs were centrifuged at 8000 rpm for 10 min, after which the precipitate was collected and vacuum freeze-dried at −40 °C to obtain a sufficient amount of powdered sample. The solid powder samples were used to perform X-ray diffraction (XRD) and X-ray photoelectron spectroscopy (XPS) analyses for characterization. X-ray diffraction (XRD) was performed using an X-ray diffractometer (X’Pert3 Powder, PANalytical, Almelo, The Netherlands) equipped with Cu Kα irradiation (λ = 1.5406 Å). XRD patterns were recorded from 10 to 90° (2θ) at a scanning speed of 2°/min at 40 kV and 40 mA. XPS analysis was performed using a Thermo K-Alpha XPS system (Thermo Fisher Scientific, Waltham, MA, USA). The survey spectra were acquired with a pass energy of 200 eV for survey scans and 50 eV for high-resolution scans, with an energy step of 1 eV.

### 2.8. Preparation and Electrochemical Testing of Fla-Pd NW-Based Biosensors

A 3.0 mm GCE was sequentially polished with Al_2_O_3_ polishing powder of different particle sizes. The electrode was ultrasonically cleaned with filtered deionized water and then air-dried. Subsequently, 10 μL of the flagella solution was dropped onto the GCE and dried at 4 °C. Next, 10 μL of the synthesized 50 μg mL^−1^ Fla-Pd NW solution was dropped onto the flagella-modified GCE and dried at room temperature.

To evaluate the electrochemical activity of the Fla-Pd NWs, the prepared working electrode was subjected to electrochemical performance testing. A conventional three-electrode system was used, consisting of a 1 cm^−2^ platinum sheet as the auxiliary electrode, the Fla-Pd NW-modified GCE as the working electrode, and an Ag/AgCl electrode as the reference electrode.

The CV tests on these samples under different treatments were performed using a workstation (CHI 760, Chenhua Instrument, Shanghai, China) in the range of −0.8 V to 1.2 V at scan rates of 50 mV s^−1^ in a 0.5 mmoL L^−1^ Fe(CN)_6_^3−^ solution with 0.1 moL L^−1^ KCl.

Electrochemical impedance spectroscopy (EIS) was performed to determine the resistance of the samples at frequencies ranging from 1 to 100,000 Hz with a Fe(CN)_6_^3−^ solution as the electrolyte.

Differential pulse voltammetry (DPV) analysis was conducted with a pulse height of 50 mV, pulse width of 500 ms, step height of 16.7 mV, step time of 500 ms, and scan rate of 4 mV s^−1^.

### 2.9. Evaluation of Sensor Reusability and Consistency Across Electrodes

The stability of the sensor upon repeated use was assessed by measuring the DPV current response at 0.5 μM of microRNA-21. The Fla-Pd-ssRNA-modified electrode was immersed in a 0.5 μM microRNA-21 solution in 0.1 M PBS buffer (pH 7.4). After each reaction, the electrode was rinsed with PBS to remove any unbound molecules. The DPV current response was recorded, and the electrode was regenerated before repeating the test for five cycles. The sensor’s reusability was evaluated by comparing the current intensity across cycles.

In a separate evaluation, four different GCE electrodes were tested for their current response to the same concentration of microRNA-21 (0.5 μM) in 0.1 M PBS buffer (pH 7.4). The DPV current response for each electrode was recorded, and the consistency and variability of current intensities were compared to assess sensor stability and reproducibility across different electrodes under identical conditions.

## 3. Results

### 3.1. Optimized Extraction and Characterization of E. coli Flagella and Fla-Pd NWs

**Figure 1:**
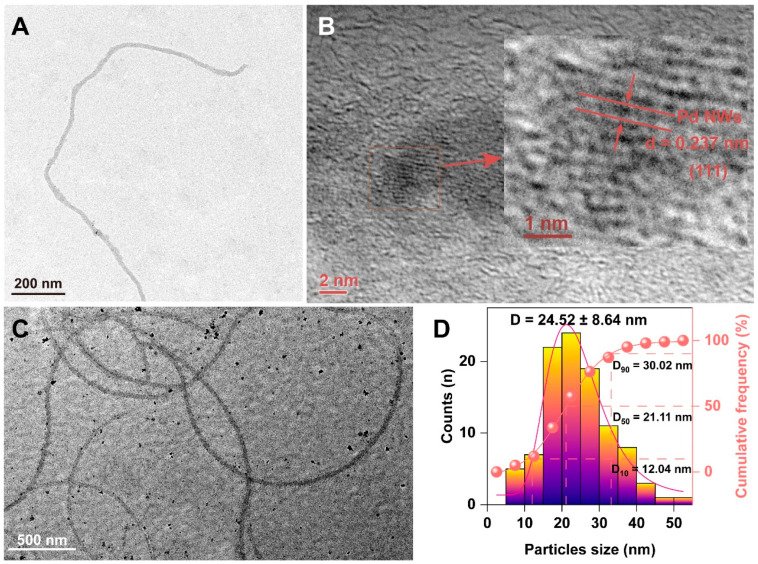
TEM image of (**A**) Fla-Pd NWs, (**B**) HRTEM image of Pd NWs, (**C**) TEM image of flagella-Pd NWs at 500 nm, and (**D**) particle size distribution.

To increase the efficiency of *E. coli* flagella extraction, we developed an optimized method implementing membrane filtration and ultrafiltration techniques. This optimized extraction process resulted in a high concentration of flagellar suspension with minimal impurities. From 6 L of bacterial culture, we isolated 1.5 mg of pure flagella solution. Despite some degree of cross-linking and regular interweaving among the flagella, probably due to S-S bonds between flagellin proteins, the flagella exhibited excellent dispersibility in aqueous solution [27]. This optimized method thus significantly improved the purity and yield of the flagella, facilitating their application in the fields of biomedicine and nanotechnology. In the high-resolution transmission electron microscopy (HRTEM) image, we observed lattice fringes (Figure 1A). Pd NWs synthesized using flagella as a template were examined at different magnifications using the electron microscope. The deposition of particles on the surface of the flagella was captured in the TEM images. The images showed the presence of well-formed nanowires that retained the original size and morphology of the flagella, which is advantageous for their biological applications. The diameter of each nanowire was about 21 nm, indicating uniformity and maintenance of the original dimensions of the flagellum. An HRTEM image of the Pd NWs is shown in Figure 1B. The lattice spacing of the Pd NWs, calculated to be 0.237 nm, corresponded to the (111) planes of octahedral palladium nanocrystals [28]. The (111) crystal face has low cytotoxicity and a promising safety profile, making these Pd NWs suitable for biomedical applications. The Pd NWs in Figure 1A were synthesized with a co-incubation time of 24 h, whereas those in Figure 1C were synthesized over a longer incubation time of 48 h, accounting for the difference in particle deposition. The Pd NWs had a spherical shape with a size distribution of 24.52 ± 8.64 nm (Figure 1C,D).

The FT-IR spectrum of the extracted surface of the flagella (Figure S1) revealed the presence of various functional groups. The peak at 3283 cm^−1^ was attributed to the N–H stretching of amide bonds, which are common in proteins [29]. This absorption band occurred probably due to the hydroxyl-containing residues of serine and threonine in the flagellin protein filaments [30]. The major band at 1637 cm^−1^ represented the amide I band component, which formed due to the C=O stretching of peptide bonds in the flagella [31]. The band at 1550 cm^−1^ corresponded to the amide II band [32]. These results indicated that the extracted flagella were rich in carboxyl (-COOH) and amino (-NH_2_) groups. These functional groups had strong coordination abilities with noble metals, suggesting that the functional groups on the surface of the flagella could act as nucleation sites for the formation of metal nanoparticles.

**Figure 2:**
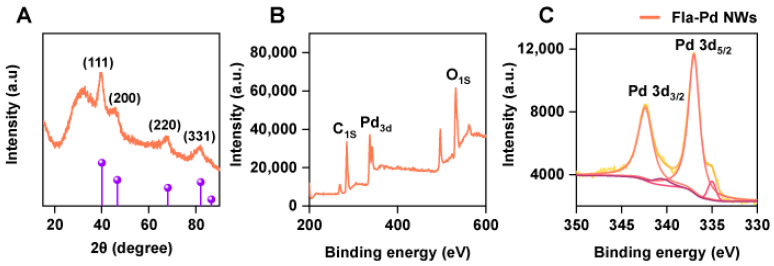
(**A**) XRD patterns of the flagella-Pd NWs, (**B**) full XPS spectrum, and (**C**) Pd XPS spectrum of the Fla-Pd NW sample.

In this study, the phase composition and crystal structure of Pd NWs synthesized using flagella templates were analyzed by XRD. The XRD diffraction patterns of the Fla-Pd NWs are shown in Figure 2A. The diffraction peaks detected at 2θ values of 39.9°, 45.8°, 68.1°, and 81.9° corresponded to the (111), (200), (220), and (331) crystal planes of palladium, respectively, indicating that the synthesized Pd NWs possess a face-centered cubic polycrystalline structure [33]. A diffraction peak was observed at 32.4°, which probably corresponded to the (101) crystal plane of PdO.

Further analysis of the Fla-Pd NW structure was conducted via XPS. The binding energies of the Pd 3d_3/2_ and 3d_5/2_ orbitals were 343.53 eV and 338.13 eV, respectively (Figure 2B,C), which corresponded to the (111) crystal plane of palladium [27].

To further elucidate the structural functionality of the Fla-Pd NWs, FTIR spectroscopy was conducted (Figure S2). The FTIR spectrum of the Fla-Pd NWs showed three functional groups (Figure S2). Notably, strong absorption peaks were observed at 1650 cm^−1^ and 1580 cm^−1^, which corresponded to the stretching vibrations of C=O in peptide bonds. These peaks indicated the presence of significant peptide bonding interactions on the Fla-Pd NW surface [34].

### 3.2. Electrochemical Properties of Fla-Pd NWs

The electrochemical activities of the Pd NWs and Fla-Pd NWs were further evaluated by constructing electrodes with these materials. CV was performed to characterize the biosensors, which revealed significant differences between the two methods. Compared to the GCE modified with Pd NWs synthesized without the flagella template, the Fla-Pd-modified GCE showed considerably higher redox peak currents and electron transfer rates for Fe(CN)_6_^3−^.

The results of the CV analysis indicated that the capacitance of the Fla-Pd NWs synthesized in this study was 3.33 times greater than that of the Pd NWs (Figure S3). The capacitance of the Fla-Pd NWs was recorded as 89.27 μF, which was significantly greater than the capacitance of the Pd NWs (26.82 μF). The higher capacitance of the Fla-Pd NWs suggested enhanced electroactivity and a superior capacity to store electrons, indicating that they perform better in electrochemical applications. This increase in performance highlighted the efficacy of the flagella template in synthesizing highly functional nanomaterials.

### 3.3. Detection of microRNA-21 and Reusability of the Fla-Pd NWs Biosensor

In this study, ssRNA probes were effectively adsorbed onto Fla-Pd NW electrodes through thiol-amino interactions. These RNA probes, which are highly complementary to microRNA-21, have high biosensor specificity and stability because of their superior RNA–RNA hybridization stability. Additionally, toluidine blue, which binds strongly to nucleic acid bases via π-π interactions, serves as an electrochemical signal amplifier. Its quasi-reversible oxidation at physiological pH allows the quantification of target microRNA sequences hybridized to the microRNA-21 probe. The cyclic voltammogram of TB with ssRNA and targeting RNA showed no significant current signal in the absence of Fla-Pd NWs, as shown in Figure S4.

**Figure 3:**
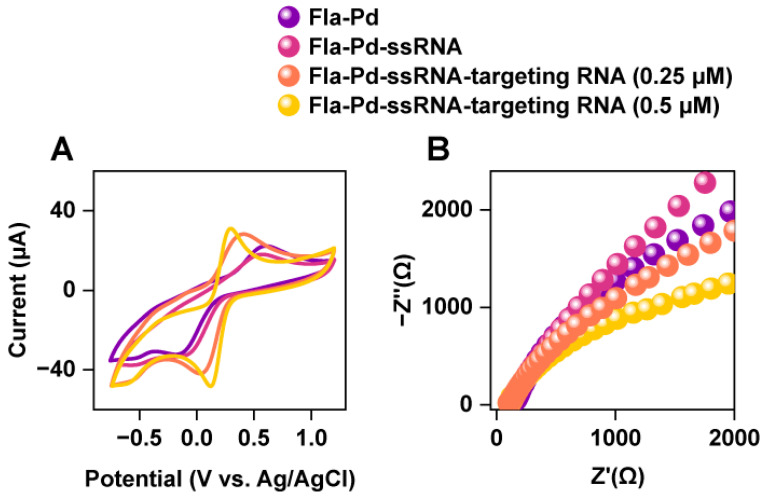
(**A**) Cyclic voltammogram of different treatment samples. Nyquist plot of (**B**) different treatment samples.

To further investigate the effect of thiolated probes on the electrochemical biosensor composed of Pd NWs, we self-assembled ssRNA capture probes onto the surface of the electrode and used CV curves to determine their detection specificity. Due to the non-conductive nature of oligonucleotides, the redox peak current and capacitance of Fla-Pd-ssRNA (0.1262 μF/μg) were significantly lower than those of Fla-Pd alone (0.1431 μF/μg) (Figure 3A). When Fla-Pd-ssRNA was immersed in 0.25 μM microRNA-21, the capacitance increased considerably (0.2122 μF/μg).

Upon increasing the concentration of the target microRNA-21 to 0.5 μM, the capacitance (0.1858 μF/μg) decreased significantly compared to the capacitance of Fla-Pd-ssRNA immersed at 0.25 μM. This suggested that as the concentration of microRNA-21 increased, the enhanced hybridization effect increased the spatial hindrance during electron transfer, resulting in a decrease in capacitance compared to that of lower concentrations of the target microRNA. However, the capacitance was still greater than that observed in the absence of the targeting microRNA. These results indicated that the binding of microRNA-21 to the ssRNA capture probes not only maintained the electron transfer efficiency but also increased the overall electrochemical performance of the biosensor.

The changes in electron transfer resistance (Rct) among different bioelectrodes were further investigated by EIS (Figure 3B). Initially, the Rct of Fla-Pd NWs without target RNA binding was measured to be 5479 Ω, which was significantly lower than the Rct recorded for Pd NWs (14,479 Ω). This decrease in resistance indicated that the binding of the flagella to the nanowires enhanced the bioelectronic transfer capability, thus facilitating more efficient electron transfer.

Upon binding with various concentrations of target RNA, a further decrease in Rct was observed, indicating an improvement in the electron transfer capability. This finding suggested that toluidine blue greatly contributed to enhancing the electron transfer ability of the bioelectrode. Specifically, at higher concentrations of target RNA, the Rct values decreased substantially, demonstrating a dose-dependent increase in electron transfer efficiency.

The combination of Fla-Pd NWs and toluidine blue improved the conductivity and significantly amplified the electrical signal. This improvement might be attributed to the synergistic effect caused by the interaction between the bioelectrode materials and the dye, which together enhanced the overall electron transfer process.

**Figure 4:**
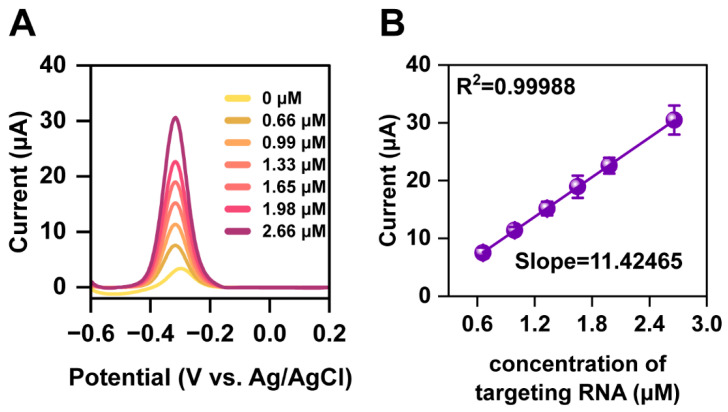
Detection of different RNA concentrations by the biosensor. (**A**) DPV response peak current and (**B**) linear fitting curve.

Based on the findings of the preceding electrochemical tests, we further investigated the current changes in the modified Fla-Pd NW bioelectrode sensor by conducting DPV for specifically labeled ssRNA at various concentrations. The peak current intensity of the biosensor exhibited a stable linear relationship with the ssRNA concentration (Figure 4A). As the concentration of ssRNA increased, the peak current intensity of the sensor also increased. The linear range of the detected peak current intensity was 0.66–1.98 μM (Figure 4B).

The findings could be attributed to an increase in the number of binding sites available for electron transfer as the concentration of ssRNA increased, which resulted in an increase in electron transfer efficiency and, thus, an increase in peak current intensity. According to the three times signal-to-noise ratio principle, the detection limit for ssRNA in this study was determined to be 0.78 μM.

**Figure 5:**
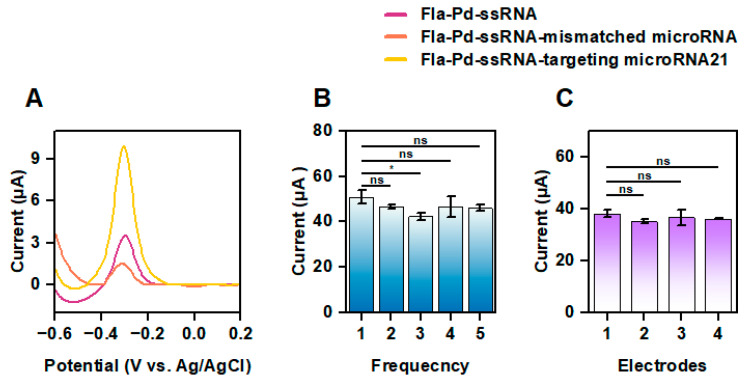
Study of the electrochemical biosensor performance of Fla-Pd NWs: (**A**) DPV peak current of different types of RNA samples, (**B**) electrochemical DPV current response after five cycles at 0.5 μM of microRNA-21, and (**C**) DPV current response of four electrodes under the same conditions at 0.5 μM of microRNA-21. ns > 0.05, * *p* ≤ 0.05, *n* = 3 for each group.

The biosensor demonstrated significant advantages, such as high sensitivity and non-toxicity (Figure 5). When the biosensor was coupled with the target RNA, the DPV peak current increased substantially compared to that of Fla-Pd NW-ssRNA. In contrast, the DPV peak current decreased when the sensor interacted with mismatched RNA. This increase in bioelectrode performance was attributed to the integration of Fla-Pd NWs, which effectively improved the electron transfer ability and, consequently, the overall sensing performance. Additionally, the biosensor maintained robust current intensity even after it was used for multiple cycles. In Figure 5B, the DPV current response of the biosensor at 0.5 μM of microRNA-21 showed minimal variation over five cycles, with only a slight difference observed between the first and second cycles. This indicated that the sensor maintained stable current intensity over repeated uses, demonstrating good reusability.

In Figure 5C, the DPV current response across four different GCE electrodes under identical conditions showed no significant differences. This consistency across electrodes confirmed the sensor’s stability and reproducibility, making it reliable for detection under varying electrode conditions.

## 4. Discussion

In this study, we described a novel approach for improving the extraction of *E. coli* flagella, which can serve as an effective template for synthesizing Pd NWs. We developed a highly efficient and pure method for flagellar extraction, which subsequently facilitated the uniform synthesis of Pd NWs. This approach not only enhances the structural integrity and functionality of the synthesized nanowires but also reveals new ways to apply them in nanotechnology and biomedicine.

The optimized extraction method for flagella used in this study has high yield and purity, which are crucial for the efficient synthesis of nanomaterials. Traditional methods of flagellar extraction are often affected by impurities and cross-contamination, which can hinder their application as biological templates in nanomaterial synthesis. The success of this method indicates its potential for scalable production, which can significantly affect the field of biomanufacturing [35].

The use of bacterial flagella as a green biological template offered several environmental benefits compared to conventional synthesis methods. Studies on green biological templates have shown that they eliminate the need for toxic organic solvents and energy-intensive procedures, which were commonly associated with traditional chemical methods. This eco-friendly approach not only reduced environmental pollution but also decreased energy consumption, aligning with green chemistry principles [36]. Moreover, biological templates could lead to more efficient material synthesis, as they naturally provided nanoscale structures that reduced the need for additional shaping or structuring steps [36].

The use of flagella as a biological template for synthesizing Pd NWs can significantly contribute to the field of nanomaterials. The Pd NWs synthesized in this study retained the morphological characteristics of the flagella, indicating that the biological structure provided a robust framework for the formation of nanowires [23]. This approach not only ensured the uniformity of the nanowires but also increased their electrochemical properties, making them suitable for advanced biosensing applications. Some studies have highlighted the challenges of maintaining structural integrity during nanomaterial synthesis, and this study addressed these challenges by using the natural properties of flagella [37].

Although the linear range (0.66–1.98 µmol/L) and LOD of 0.78 µmol/L for the sensor were not at par with some high-sensitivity methods, our approach offered several unique advantages. First, the use of bacterial flagella as a biological template introduced an innovative strategy, offering a novel pathway for synthesizing metal detectors based on bacterial subunits. This method expands the possibilities for nanomaterial design, allowing for greater diversity and functionality. Second, the synthesis process is eco-friendly; it avoids the use of toxic reagents and adheres to the principles of green chemistry. This approach not only simplifies the synthesis process but also offers a safer alternative for practical applications. Third, the bioderived template enhances the biocompatibility of the synthesized nanomaterials, decreasing toxicity and improving reliability in real-world applications.

The findings of this study regarding the functional groups present on the surface of the flagella and their interaction with palladium ions also provided valuable insights into the mechanisms underlying nanowire synthesis. The presence of thiol, carboxyl, and amino groups on the surface of the flagella facilitated strong coordination with noble metals, greatly contributing to the nucleation and growth of the metal nanoparticles. This discovery was consistent with the findings of studies that emphasized the importance of surface functionalization in regulating nanoparticle synthesis [38,39,40,41,42].

The enhanced electrochemical performance of the Fla-Pd NWs, as determined by the CV and EIS results, highlighted the greater electron transfer capabilities of the synthesized nanowires. The significant increase in capacitance and reduction in Rct compared to those of nontemplate-synthesized Pd NWs showed that using flagella as a template is an efficacious strategy [43]. This finding is particularly relevant in the context of biosensing applications, where the sensitivity and specificity of the sensor are extremely important. The integration of thiolated ssRNA probes further enhanced the functionality of the biosensor, demonstrating a synergistic effect caused by the interaction between the biological template and the nanomaterial [44].

Compared to existing methods of Pd NW synthesis, the use of flagella templates in this study represents a significant advancement. These features make our method valuable for applications in situations where conventional synthesis techniques may pose environmental or biocompatibility challenges. Traditional chemical and physical methods for synthesizing Pd NWs often require harsh conditions and complex processes that can limit their applicability in biological systems. In contrast, our biotemplate approach not only simplified the synthesis process but also resulted in the formation of nanowires with superior biocompatibility and functionality. These findings suggested that Fla-Pd NWs are promising materials for a wide range of applications, from catalysis to targeted drug delivery.

## 5. Conclusions

This study successfully developed an optimized method for extracting *E. coli* flagella, resulting in high-purity flagella suspensions that served as templates for synthesizing Pd NWs. The synthesized Pd NWs retained the structural integrity of the flagella and exhibited enhanced electrochemical properties, making them suitable for biosensing applications. In the future, the scalability and cost-effectiveness of this method would be key to its widespread application. The use of biotemplates reduced the need for toxic reagents, aligned with green chemistry principles, and enhanced the sustainability and environmental friendliness of the synthesis process.

Additionally, this method opened new avenues for future research. Exploring different biological templates could further diversify the types of nanomaterials produced, expanding applications in fields such as medicine and energy storage. Investigating the cost-effectiveness and industrial-scale implementation of this method would enhance its commercialization potential. In conclusion, this innovative approach not only provided a novel pathway for synthesizing palladium nanowires but also paved the way for greener and more sustainable nanomaterial production.

## Data Availability

All data needed to evaluate the conclusions in the paper are present in the paper and/or the Supplementary Materials.

## Supplementary Materials

The following supporting information can be downloaded at: https://www.mdpi.com/article/10.3390/biology13120960/s1, Figure S1: FT-IR pattern of flagella; Figure S2: FTIR profile of Fla-Pd NWs; Figure S3: Comparison of CV curves of different electrodes; Figure S4: The cyclic voltammogram of TB with ssRNA and targeting RNA; Table S1: Nucleotide sequences required in this experiment; Table S2: Buffer formulations required in this experiment.
