# Supplementary material for: Synthesis of Palladium Nanowires on Flagella Template for Electrochemical Biosensor Detection of microRNA-21
Source: Biology (Basel). 2024 Nov 22;13(12):960. doi: 10.3390/biology13120960 (PMC11727094; doi:10.3390/biology13120960)
Supplement: Supplementary file 1 [file biology-13-00960-s001.zip › biology-3273550-supplementary.pdf]

Synthesis of palladium nanowires on flagella template for electrochemical biosensor detection of microRNA-21

Authors:

Kuo Yang[1,**], Jueyu Wang[1,**], Ying Zhang[1], Daizong Cui[1,*], Min Zhao[1,*]

Affiliations:

[1]College of Life Science, Northeast Forestry University, Harbin, 150000, China.

[*]Corresponding author. Daizong Cui: daizongcui@nefu.edu.cn; Min Zhao: zhaomin@nefu.edu.cn.

**These authors contributed equally: Kuo Yang, Jueyu Wang.

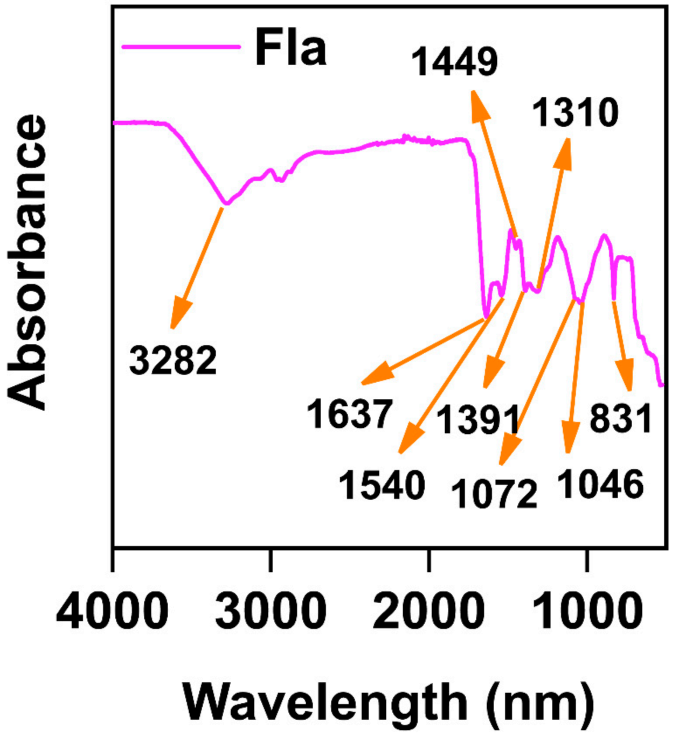

Figure. S1 FT-IR pattern of flagella

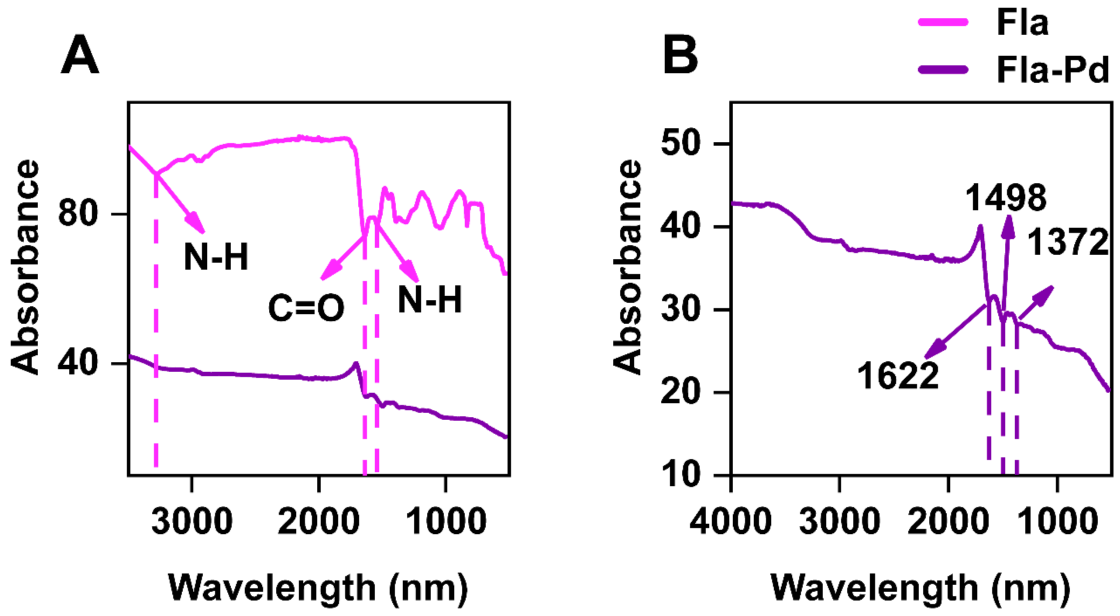

Figure. S2 FTIR profile of Fla-Pd NWs

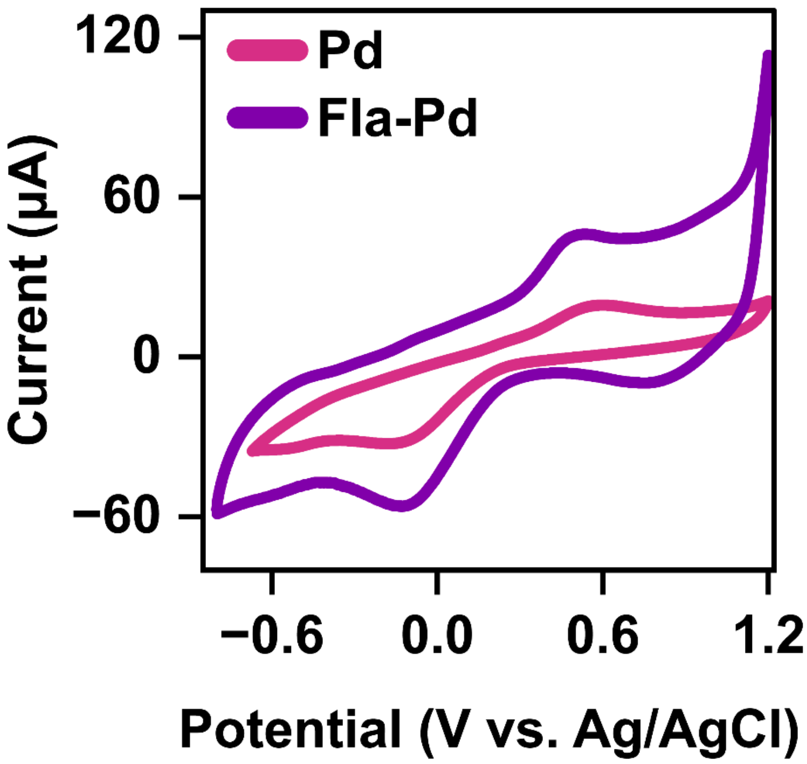

Figure. S3 Comparison of CV curves of different electrodes

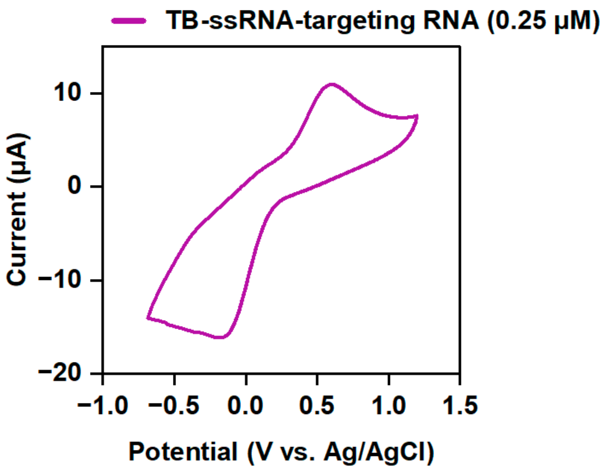

Figure. S4 The cyclic voltammogram of TB with ssRNA and targeting RNA

Table S1 Nucleotide sequences required in this experiment

Reagant	Sequence
Probe ssRNA	5’-SH-UCA ACA UCA GUC UGA UAA GCU A-3’
Targeting microRNA-21	5’- UAG CUU AUC AGA CUG AUG UUG A-3’
Mismatched microRNA	5’-UAG CUU AUC GGA GUG AUG UUG-3’

Table S2 Buffer formulations required in this experiment

Reagant	Composition
ssRNA fixation buffer	1.0 M NaCl solution prepared in 10 mM pH 5.4 K_2HPO_4-citric acid buffer
Hybridization Buffer	Mixture of 10 mM trisodium citrate and 0.15 M NaCl prepared in purchased SSC (1×) buffer
Electrolyte Buffer	0.15 M NaCl solution prepared in 0.02 mM PBS buffer

Note: All solutions used in this experiment were treated with diethyl pyrocarbonate (DEPC). Centrifuge tubes, glassware, and pipette tips were sterilized using high-temperature and high-pressure autoclaving, and treated with DEPC before use.
